# DNA Methylation: A Target in Neuropathic Pain

**DOI:** 10.3389/fmed.2022.879902

**Published:** 2022-07-07

**Authors:** Wei Jiang, Xuan-Yu Tan, Jia-Ming Li, Peng Yu, Ming Dong

**Affiliations:** ^1^Department of Neurology and Neuroscience Center, The First Hospital of Jilin University, Changchun, China; ^2^Department of Neurosurgery, The First Hospital of Jilin University, Changchun, China; ^3^Department of Emergency, The First Hospital of Jilin University, Changchun, China; ^4^Department of Ophthalmology, The Second Hospital of Jilin University, Changchun, China

**Keywords:** neuropathic pain, DNA methylation, gene suppression, epigenetic, treatment

## Abstract

Neuropathic pain (NP), caused by an injury or a disease affecting the somatosensory nervous system of the central and peripheral nervous systems, has become a global health concern. Recent studies have demonstrated that epigenetic mechanisms are among those that underlie NP; thus, elucidating the molecular mechanism of DNA methylation is crucial to discovering new therapeutic methods for NP. In this review, we first briefly discuss DNA methylation, demethylation, and the associated key enzymes, such as methylases and demethylases. We then discuss the relationship between NP and DNA methylation, focusing on DNA methyltransferases including methyl-CpG-binding domain (MBD) family proteins and ten-eleven translocation (TET) enzymes. Based on experimental results of neuralgia in animal models, the mechanism of DNA methylation-related neuralgia is summarized, and useful targets for early drug intervention in NP are discussed.

## Introduction

The latest and now widely accepted definition of neuropathic pain (NP) is pain caused by an injury or a disease of the somatosensory system (1). NP is a chronic disease with complex clinical symptoms, poor prognosis, and an increasing disease burden. Most importantly, treatment options are extremely limited, and some patients develop resistance to drugs of opioid analgesics (2). Peripheral nerve injury or disease can cause a series of NP symptoms including spontaneous pain, hyperalgesia, and allodynia. Allodynia refers to pain caused by stimuli that do not usually cause pain, whereas hyperalgesia refers to increased pain caused by stimuli that cause pain (3). Although distinct neuropathic syndromes induce pain, their clinical symptoms are similar. NP is divided into peripheral and central NP based on the pathogeny. Cellular and molecular changes in NP are associated with different pain pathways, among which epigenetic studies primarily focused on peripheral nerves, dorsal root ganglion (DRG), and dorsal horn (4).

**Figure 1:**
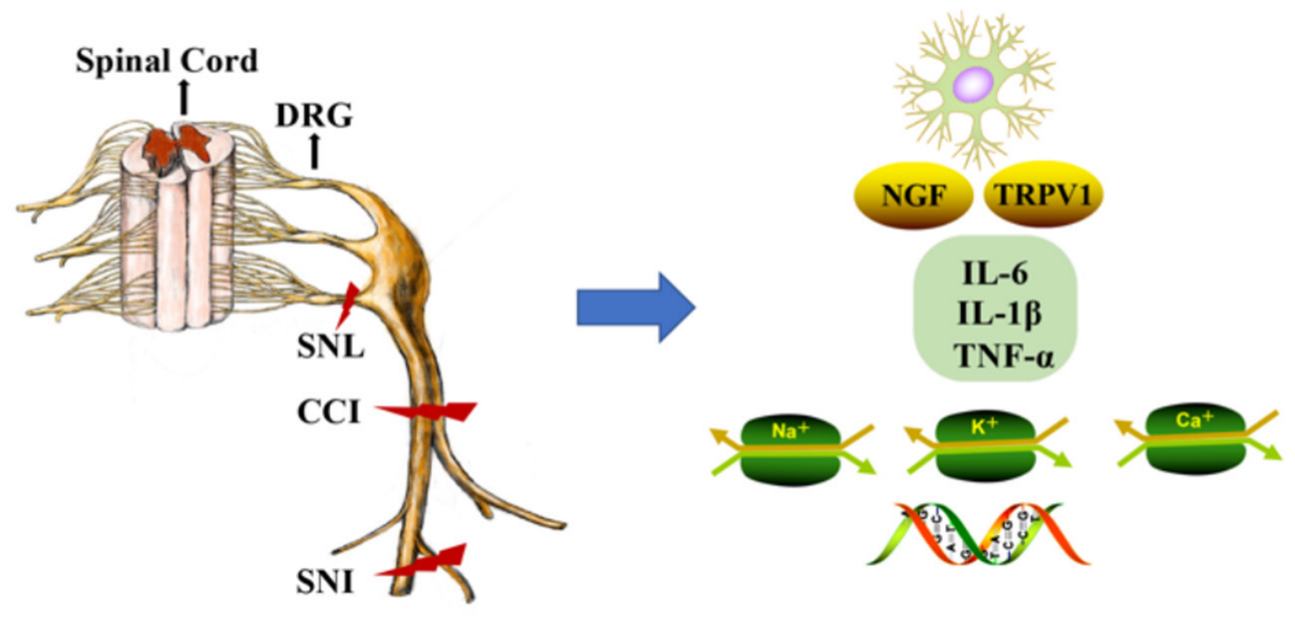
DNA methylation in the mechanism of neuropathic pain (NP). The dorsal root ganglion (DRG) and spinal cord produce glial cell responses, stimulating inflammatory cytokines, nerve growth factors, gene expression, and ion channels under the environment of peripheral afferent fiber injury. CCI, chronic constriction injury; SNL, spinal nerve ligation; SNI, spared nerve injury; DRG, dorsal root ganglion; NGF, nerve growth factors; Na^+^, Na^+^ channels; K^+^, K^+^ channels; Ca^+^, Ca^+^ channels.

The DRG is located at the junction of the peripheral and central nervous systems (5). In peripheral nerve injury, nociception is conveyed *via* primary sensory neurons in the DRG and back to secondary sensory neurons (6). It is well-known that neuropathic pain is related to hyperexcitation and internal firing of DRG neurons. There are two types of DRG neurons, type A and type B. Type A DRG neurons are large and are responsible for touch, vibration, and proprioception, and type B neurons are small and are responsible for nociception (7). DRG generates the fibers that convey information, including the activation of nociceptors from the skin, muscles, and joints to the spinal cord. With continuous nociceptive input, central sensitization of the spinal cord (i.e., increased reactivity of nociceptive neurons in the central nervous system to normal or subliminal afferent signals) plays an important role in pain perception (8). In response to peripheral afferent tissue injury, signals from the DRG and spinal cord activate glial cells, increase the expression of proinflammatory factors (IL-6, IL-1β, and TNF-α), increase the expression of receptors including nerve growth factor and TRPV1, alter gene expression, and decrease the expression of ion channels (i.e., sodium, voltage-gated potassium, and calcium channels; Figure 1) (9). Notably, voltage-gated potassium channels play an important role in inducing spontaneous ectopic discharge, the hyperexcitability of neurons, and neuropathic pain-like symptoms, which are described in the following sections.

Epigenetic processes include DNA methylation, covalent histone acetylation, and non-coding RNA expression (10). DNA is wrapped around a histone octamer consisting of dimers of histones H3, H4, H2A, and H2B. Cells regulate gene expression and the structure and function of chromatin through post-transcriptional modification of the N-terminal histone tails of nucleosomes (11, 12). Histone acetylation occurs on lysine residues and is catalyzed by the histone acetyl transferase (HAT) family, leading to transcriptional activation. Deacetylation is performed by the histone deacetylase family of enzymes (HDACs) and is involved in transcriptional inhibition (13). Methylated amino acid residues determine the inhibition or activation of gene transcription. For example, methylation of Lys9 or Lys27 of histone H3 is usually associated with gene suppression, while methylation of Lys4, Lys36, or Lys79 of H3 is usually associated with gene activation (14). MicroRNAs (miRNAs) are endogenous, non-coding functional RNAs that range from 19 to 24 nucleotides in size. They bind target mRNAs, inhibiting translation and leading to the downregulation of target proteins (15). Emerging evidence has indicated that histone acetylation and deacetylation, DNA methylation, and the regulation of miRNA are closely related to NP (16–20).

NP is involved in the activation of glial cells, the triggering of inflammatory cascades, abnormal neuronal firing, and ion channel imbalance in the central and peripheral nervous systems. Epigenetics can modulate pain responses by regulating inflammation *via* ion channels, receptors, and neurotransmitters. A growing number of studies have demonstrated that DNA methylation, histone acetylation, deacetylation, histone methylation, and miRNAs regulate pain through inflammatory responses (19–25). These studies suggest that harmful stimuli drive the activation of glial cells and are involved in epigenetic modification of NP. Ion-channel imbalances including sodium channels and voltage-gated calcium channels, often accompanied by spontaneous ectopic discharge and hyperexcitation, contribute to the occurrence of NP in DRG and spinal cord neurons. Furthermore, increasing evidence suggests that epigenetic modifications contribute to peripheral nerve injury by modifying ion channel status, and histone acetylation and methylation have been shown to reduce pain responses *via* ion-channel regulation (17, 26, 27).

As a more stable epigenetic modification, DNA methylation can silence or downregulate promoters or enhancers (28). Such distinct gene expression profiles could influence pain and analgesia. In recent years, some studies have demonstrated that DNA methylation is associated with the pathology of NP (29, 30). Although studies on the mechanism of DNA methylation account for an increasing proportion in NP research, the findings remain limited. In this review, we systematically describe DNA methylation and demethylation, briefly introduce the possible mechanism underlying DNA methylation-induced NP from the perspective of studies with experimental animal NP models, and aim to reveal a reliable future therapeutic target for NP.

## DNA Methylation and NP

### DNA Methylation, DNMTs, and Methyl-CpG-Binding Domain (MBD) Proteins

DNA methylation is important for regulating tissue-specific gene expression and may affect gene activity variably in different genomic regions. DNA methylation enables chromatin to maintain its inactive state in the following two cases: (i) silencing elements potentially harmful to DNA, such as transposons, viral DNA, and genes that should not be expressed; and (ii) avoiding the binding of transcription factors to specific sites in promoter regions or even by allowing transcription repressor binding (31). In mammals, DNA methylation is catalyzed by DNA methyltransferases (DNMTs), which predominantly add methyl groups from the S-adenosyl-l-methionine to the carbon-5 position of cytosine bases [5-methylcytosine (5mC)] mainly located at cytosine-phosphate-guanosine (CpG) islands (32, 33). Of note, the promoters of most genes, especially housekeeping genes, are located in CpG islands (34). CpG islands, especially those associated with promoters, are highly conserved between mice and humans, suggesting that these regions have important functions. Peripheral nerve injury reduces DNA methylation in the prefrontal cortex (PFC) and amygdala. The overall methylation of PFC correlates with symptom severity (35). Promoter methylation of the extracellular matrix protein gene *SPARC* is increased in cases of chronic lower back pain in humans and mice (36). Therefore, DNA methylation-mediated regulation of the pain-related genes in peripheral tissues and the central nervous system could be involved in the development and maintenance of NP.

The DNMT family includes DNMT1, DNMT2, DNMT3a, and DNMT3b (37–40). The main function of DNMT1 is to maintain established DNA methylation signatures in the genome and repair DNA methylation, and it is also known as maintenance methyltransferase (41). DNMT3a and DNMT3b can reversibly methylate unmethylated DNA and are, thus, classified as *de novo* methyltransferases (38, 39). The expression of DNMT1, DNMT3a, and DNMT3b in adult DRGs is upregulated in the NP rat model (42). DNMTs inhibit the increase in the methylation of the Mu opioid receptor (MOR) gene and prevent the decrease in MOR expression in the DRG, thereby improving morphine analgesia (43). Nerve injury significantly upregulates DNMT3a, increasing methylation of the spinal MOR gene promoter, and decreasing the expression of the MOR protein (44).

**Figure 2:**
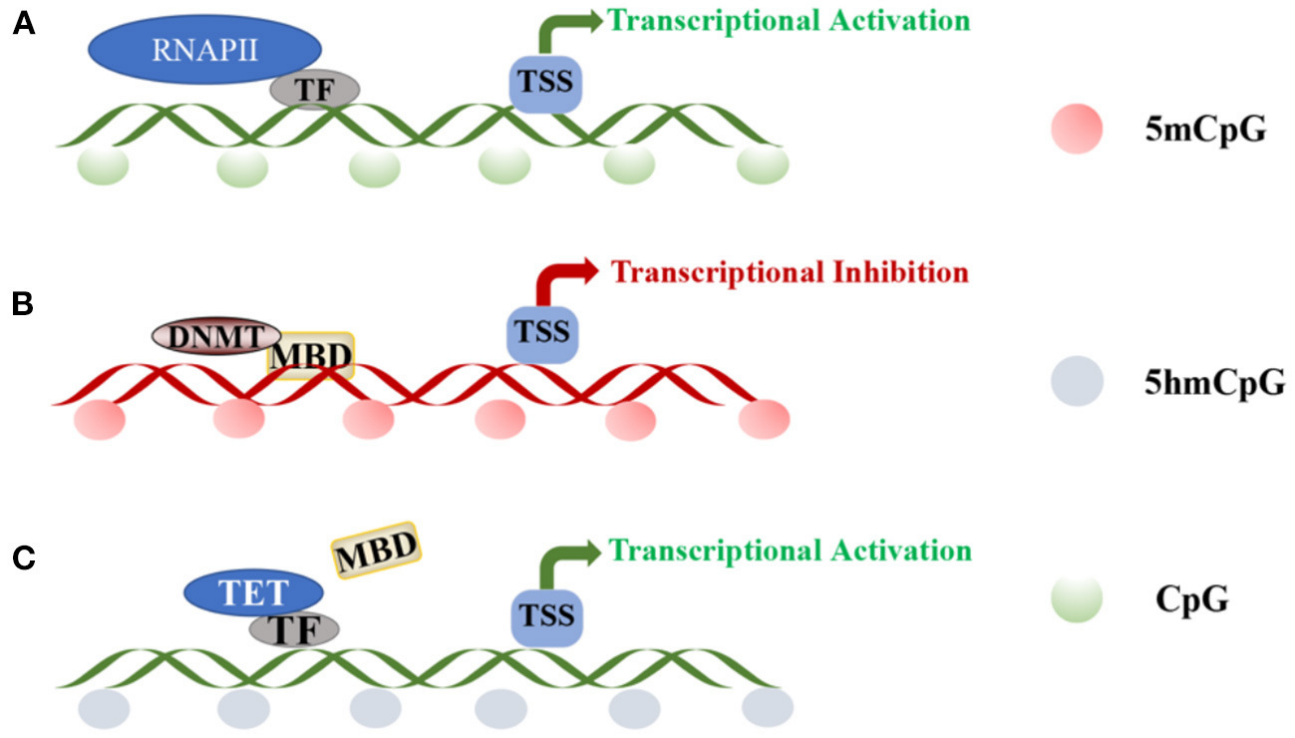
DNA methylation and demethylation: **(A)** TF and RNAPII bind to the promoter region of the gene, activating transcription at unmethylated promoters. **(B)** CpG island methylation is mediated by DNA methyltransferases and induces DNA methylation through MBD proteins. **(C)** Transcription factors recruit TET enzymes to specific sites for regulating local DNA demethylation.TF, transcription factor; RNAPII, RNA polymerase II; TSS, transcriptional start site; DNMT, DNA-methyltransferases; MBD, methyl-CpG-binding domain; TET, ten-eleven translocation.

DNA methylation is mediated by three separate families of proteins—MBD, UHRF, and zinc finger proteins (45). Of these three protein families, MBD proteins are the most well-studied, especially in the context of revealing their roles in pain. In the absence of peripheral nerve injury, transcription factors (TFs) and RNA polymerase II bind to gene promoters, activating transcription. The methylation of CpG islands disrupts the binding of TFs, recruiting DNMT-mediated MBD proteins to silence gene expression (Figures 2A,B) (45, 46). The MBD family includes methyl-CpG-binding protein 2 (MeCP2) and MBD1-6 (47, 48), wherein MeCP2 primarily functions as a transcriptional repressor. MeCP2 was downregulated in a rat neuropathic pain model, with concomitant changes in the expression of HDAC1 and HDAC2 (49). Furthermore, nerve injury upregulates MeCP2 in the DRG, and the downregulation of MOR in the DRG is closely related to the increase in the expression of MeCP2. For example, MeCP2 knockout restores the expression of MOR in damaged DRGs and enhances the analgesic effect of morphine (50). Thus, DNA methylation is a complex epigenetic process, and further studies on the role of MeCP2 in NP are warranted.

### DNMTs and NP

DNMTs, which play a key role in “reading DNA methylation,” have been studied in recent years with respect to their roles in NP. As a non-nucleoside (small molecule) DNMT inhibitor, RG108 blocks the active sites of DNMTs (51), and some studies have shown that it can relieve pain. K2p1.1 was the first K2P channel identified in mammals, and its expression is significantly downregulated after peripheral nerve injury and may lead to increased neuronal excitability (52, 53). Mao et al. (29) demonstrated that paclitaxel injection downregulated K2P1.1 in the DRG. Using a whole-cell current clamp, these authors demonstrated that neuronal excitability increased when K2P1.1 was knocked out. They also explored the underlying mechanism and found that K2p1.1 downregulation depends on the upregulation of DNMT expression. This suggested that DNA methylation may be involved in paclitaxel-induced downregulation of *K2p1.1* mRNA in the DRG. The level of DNMT3a was increased in the DRG after injection of paclitaxel and, moreover, RG108 might significantly block paclitaxel-induced mechanical allodynia. Two other studies have investigated the role of RG108 as a DNMT inhibitor. Sun et al. (54) found that RG107 increased the expression of DNMT1 in the DRG and that it did so *via* the activation of the transcription factor cAMP response element-binding protein (CREB), causing DNA methylation of the *Kcna2 (*encoding Kv1.2*)* promoter, thereby reducing Kv1.2 expression and promoting pain. Decreased Kv1.2 expression reduced the total voltage-gated potassium current, depolarized the resting membrane potential, and induced the spontaneous ectopic discharge and hyperexcitability of neurons and neuropathic pain-like symptoms. Furthermore, RG108 administration or *DNMT1* knockout reduced pain and allodynia caused by nerve damage. Specifically, *DNMT1* knockout prevented neuronal hyperexcitability in the injured DRG. Another study revealed an increase in DNMT3a expression in a chronic constriction injury (CCI) model, and that MOR methylation plays an important epigenetic role in NP. DNMT3a binds to the *MOR* promoter, inhibits its transcription, reduces its mRNA and protein expression levels, and causes pain (44). The DNMT inhibitor RG108 was found to significantly block the increase in the methylation of the *MOR* promoter, consequently upregulating MOR expression to attenuate NP.

The nucleoside DNMT inhibitor 5-azacytidine prevents the resolution of a covalent reaction intermediate, which leads to DNMT being trapped and inactivated in the form of a covalent protein–DNA adduct (55, 56). The link between 5-azacytidine and NP was subsequently further clarified, showing that nerve damage leads to an increase in DNMT3a expression, a decrease in miR-214-3p expression, and triggers colony-stimulating factor-1 (CSF1) overexpression (57). The DNMT inhibitor zebularine was also found to significantly reduce methylation of the miR-214-3p promoter, leading to an increase in miR-214-3p expression in the ipsilateral dorsal horn and a decrease in CSF-1 content, which further alleviated the pain behavior of rats after spinal nerve ligation (SNL).

Small interfering RNAs (siRNAs) and short hairpin RNAs (shRNAs) are potential epigenetic targets. Unlike DNMT inhibitors, their sequences can be designed for a specific target gene. Opioids, the gold standard for NP treatment, have unsatisfactory analgesic effects, partly due to the downregulation of opioid receptors in DRG neurons. DNMT3a reportedly inhibited expression of *Oprm1* and *Oprk1* and their respective proteins, namely, MOR and kappa-opioid receptor (KOR), in the DRG. Microinjection of shRNA-DNMT3a was found to increase the levels of opioid receptors and relieve pain (30). However, in another study, the expression of DNMT3b was markedly downregulated after SNL injury, leading to demethylation of the *GPR151* promoter, thereby promoting the binding of the transcription factor KLF5 to the *GPR151* promoter to further increase GPR151 expression. The administration of siRNA-DNMT3b further increased the GPR151 level and exacerbated pain. In contrast, DNMT3b overexpression reduced pain (58). The authors of this study showed that SNL-induced NP could decrease DNMT3b expression, and the administration of a lentivirus-carrying DNMT3b could relieve pain. They further confirmed that DNMT3b might lead to the demethylation of the *CXCR3* promoter, further increase the binding of C/EBPa and CXCR3, promote transcription and expression of *CXCR3*, and, thereby, induce NP (59).

DNMTs and their inhibitors appear to have varying effects on NP; DNMTs do not uniformly increase or decrease NP but rather act as a methylation tool that affects NP by increasing or decreasing the expression of specific genes. DNMTs may combine with gene promoters, leading to the methylation or demethylation of genes, consequently exacerbating or relieving NP. The combination of DNA methylation or demethylation enzymes with specific gene promoters is now recognized as a mode of NP treatment in the form of methylation. Thus, understanding the methylation or demethylation of the corresponding gene promoters caused by DNMT can provide new guidance for the treatment of NP. Administering gene-specific siRNA or lentiviral therapy is a prospective treatment option in the future.

### MBD and NP

Thus far, research on NP has mostly focused on MeCP2 from the MBD protein family. In the SNI and Complete Freund's Adjuvant (CFA) model, MeCP2 was found to be overexpressed in neurons and was downregulated in glial cells (49). MeCP2 was also reported to be expressed in all dorsal horn neurons of the adult spinal cord (60). Notably, intrathecal 5-azacytidine administered *via* spinal injection significantly inhibited the increase in global DNA methylation and MeCP2 expression in the spinal cord (61). In another study, the expression of MeCP2 in the DRG was upregulated after spared nerve injury (SNI) surgery, which mediated the upregulation of brain-derived neurotrophic factor (BDNF) expression and led to pain. Moreover, microRNAs can decrease the expression level of MeCP2, thereby inhibiting BDNF expression and reducing pain (62). *Mecp2*-null mice also exhibited decreased BDNF levels in the DRG and decreased pain sensitivity. Central sensitization refers to nociceptor inputs that trigger an increase in the excitability of neurons in the central pain pathway and the prolongation of synaptic potency (63). Accumulating evidence indicates that in addition to activity-dependent synaptic plasticity, changes in gene transcription contribute to the maintenance of central sensitization (37). As a sensory regulator in the nociceptive pathway, BDNF affects central sensitization (64). In CCI model rats, DNMT3a, DNMT3b, and MeCP2 expression levels increased while the MBD2 expression level decreased in the lumbar spinal cord. The authors of this study also determined that *GAD-1* promoter methylation reduced the level of GAD-67 protein, which is the main inhibitor of γ-aminobutyric acid (GABA) synthetase (65). However, they did not further explain the connection between gene methylation, DNMT, and the MBD family. Therefore, the subtype changes between DNMT and MBD warrant further study.

In contrast to these conclusions, Zhang et al. (66) reported that MeCP2 can reduce pain sensitivity. These authors attenuated mechanical and thermal pain sensitivity through *MeCP2* overexpression, rather than administering MeCP2 inhibitors. The mechanism was found to involve the CREB/miR-132 signaling pathway in the spinal cord. In a study of the relationship between MBD and NP, Mo et al. (67) demonstrated that MBD1 deficiency in the DRG triggered a reduction in pain hypersensitivity following peripheral nerve injury; MBD1 recruits DNMT3a to the promoters of the *Oprm1* and *Kcna2* genes in DRG neurons, inhibiting MOR and Kv1.2 expression and inducing NP. Furthermore, both M*bd1* knockout and intrathecal administration of siRNA-*Mbd1* reduced the sensitivity of mice to pain.

DNA methylation is a highly complex process. Both methylation and demethylation regulate gene expression by inhibiting or activating genes, respectively. The differences in MeCP2 function previously discussed may be caused by its effect on different genes. Another reason for the contradictory effects of MeCP2 in NP models may be related to the temporal properties of the protein.

## DNA Demethylation

### DNA Demethylation and TET

A balance in DNA methylation and demethylation should be achieved in neurons. In different biological studies, DNA demethylation has been verified to be both an active and passive process. Active demethylation refers to the removal or modification of methyl groups from 5mC during certain enzymatic processes (68). Notably, along with being an intermediate form of DNA demethylation, 5-hydroxymethyl cytosine (5hmC) is an epigenetic marker enriched within promoters and gene bodies (69). The continuous reaction of ten-eleven translocation (TET) enzyme initiates DNA demethylation, and the conversion of 5mC to 5hmC is divided into two pathways—oxidation and deamination. TET proteins are large (~180–230 kDa) multidomain enzymes and include TET1, TET2, and TET3 (70), with high neuronal and low glial expression (71). Purified TET enzymes were found to modify oligonucleotide substrates containing 5mC through oxidation, and the product was authenticated as 5hmC in CpG regions (72–74). The three TET enzymes share a conserved C-terminal domain and a less-conserved N-terminal domain (75). Additionally, TET1 and TET3 may have similar functions, mainly regulating 5hmC levels at gene promoters and transcription start sites, while TET2 mainly regulates 5hmC levels in the gene body (71, 76). Therefore, although the TET family can generate 5hmC, they may regulate the expression of 5hmC at different cell sites or at different developmental stages and at different genomic sites. Several non-enzymatic proteins, such as TF, could regulate local DNA demethylation in a sequence-specific manner by recruiting TET enzymes to specific sites (Figure 2C) (77–79). Significantly high levels of 5hmC are found in adult neuronal cells (80), and 5hmC is not only an intermediate for DNA demethylation but also acts as a stable epigenetic marker, which is enriched in genomes, promoters, and transcription factor-binding sites, potentially affecting gene expression. Pan et al. (81) showed that TET1 and TET3 levels increase significantly in the spinal cord with increased 5hmC content of the whole genome. Therefore, 5hmC is a key intermediate that activates the demethylation pathway. This discovery provides a crucial clue to enrich our understanding of the mechanism of DNA demethylation.

### TET and NP

TET3 was suggested to be the main driving factor for the upregulation of 5hmC in the DRG after nerve injury (82). Wu et al. (83) further showed that SNL surgery could trigger methylation of *Oprml1* and *Kcna2*, thereby increasing the amounts of 5mC with a corresponding decrease in the level of 5hmC, thus reducing the corresponding MOR and Kv1.2 protein levels and ultimately causing pain. In contrast, overexpression of DRG TET1 can block the methylation of these two genes and increase the 5hmC level, thereby reducing pain. The same authors also confirmed that overexpression of TET by DRG microinjection of the herpes simplex virus-TET1 could improve morphine analgesia and prevent morphine tolerance under NP conditions (83). However, Hsieh et al. (73) demonstrated that TET1 expression was enhanced after SNL surgery. Similar to the results of previous studies, the level of 5hmC in the dorsal horn of the ipsilateral spinal cord increased in proportion to the enhanced TET1 expression by SNL. The level of 5hmC, the excitability of dorsal horn neurons, and pain could be improved and alleviated after intrathecal siRNA-TET1 administration. Furthermore, SNL increased the binding of TET1 to the *Bdnf* promoter and increased the 5mC/5hmC transformation mediated by TET1 at the CpG site of the *Bdnf* promoter. These effects could be reversed by spinal cord-targeted injection of siRNA-TET1 (73). The same group subsequently showed that NP is related to TET1-mediated demethylation (84); melatonin was found to reverse TET expression, mGluR5 promoter demethylation, and pain hypersensitivity induced by *Tet1* gene transfer. We summarize the key studies focusing on the links between DNMT, MBP, and TET proteins with NP in Table 1.

**Table 1:** Roles of DNMT, MBD, and TET proteins in neuropathic pain (NP) in rodent models.

**Pain model**	**DNMT involved**	**Tissue**	**Target genes (Positive control ↑ and negative control ↓)**	**Inhibition or overexpression**	**Nociceptive behavior response to inhibitors**	**Reference**
Paclitaxel-induced	DNMT3a	DRG	K2P.1.1 ↓	RG108	Thermal ↓ Mechanical ↓	(52)
SNL	DNMT3a	DRG	MOR and KOR ↓	ShRNA-DNMT3a	Thermal ↓	(30)
SNL/CCI	DNMT1	DRG	Kv1.2↓	RG108	Thermal ↓ Mechanical ↓ Cold ↓	(54)
CCI	DNMT3a	Spinal cord	MOR ↓	RG108	Thermal ↓	(44)
SNI	DNMT3a	DRG	CFS1 IL-6 ↓	Zebularine	Thermal ↓ Mechanical ↓	(57)
SNL	DNMT3b	Spinal cord	GPR151↓	siRNA-DNMT3b	Thermal ↑ Mechanical ↑	(58)
SNL	DNMT3b	Spinal cord	CXCR3 ↑	LV-*Dnmt3b*	Thermal ↓ Mechanical ↓	(59)
SNL	MBD1	DRG	MOR and KV.1.2 ↓	MBD1–/– MBD1-siRNA	Thermal ↓ Mechanical ↓ Cold ↓	(67)
CCI	MECP2	Spinal cord	/	5-Azacytidine	Thermal ↓ Mechanical ↓	(61)
SNI	MECP2	DRG	BDNF↑	MeCP2-null	Mechanical ↓	(62)
CCI	MECP2 and MBD2	Spinal cord	GAD679(MeCP2 ↓, MBP2 ↑)	/	/	(65)
SNI	MECP2	Spinal cord	p-CREB ↓	Overexpressing MeCP2	Thermal ↓ Mechanical ↓	(66)
SNI	TET3	DRG	/	/	/	(82)
SNL	TET1	DRG	Oprml1 and Kcna2 ↑	HSV-TET1	Thermal ↓ Mechanical ↓	(83)
SNL	TET1	Spinal cord	mGluR5↓	Melatonin	Thermal ↓ Mechanical ↓	(84)
SNL	TET1	Spinal cord	BDNF ↓	siRNA-TET	Thermal ↓ Mechanical ↓	(73)

*CCI, chronic constriction injury; SNL, spinal nerve ligation; SNI, spared nerve injury; siRNA, small-interfering RNA; shRNA, short hairpin RNA; DRG, dorsal root ganglion; DNMT, DNA methyltransferases; MBD, methyl-CpG-binding domain; TET, ten-eleven translocation; LV, lentivirus; HSV, herpes simplex virus*.

Overall, these results suggest that TET-based treatment of NP remains complex because although TET1 overexpression can alleviate pain, TET reduction can also relieve pain. Moreover, similar to DNMT, the genes that TETs combine with also vary. Furthermore, the demethylation of other genes promoted by TET1 overexpression or TET inhibition under NP conditions cannot be excluded. Thus, treating NP with TET requires more in-depth research and extensive trials.

## Challenges and Perspectives

NP is a complex disease with multiple pathologies. DNA methylation could act as a trigger, a downstream response mechanism, or could play a role in both processes. Epigenetic adaptations lead to chronic increases in hyperalgesia and allodynia; therefore, new treatment strategies that address NP are necessary (85). The DNMT inhibitors 5-azacitidine and decitabine are approved for treating myelodysplastic syndrome (86, 87). As a DNMT inhibitor, decitabine was approved in May 2006 for the treatment of myelodysplastic syndromes (MDS) and chronic myelogenous leukemia. Adverse reactions, such as hyperbilirubinemia, pneumonia, and constipation, appeared in phase 3 of the trial during treatment of MDS (88); however, other DNMT inhibitors have not been clinically applied. Therefore, many clinical trials are required to evaluate if they have the same side effects when applied to NP patients. Furthermore, not all drugs are tissue-specific. Thus, epigenetic treatment of NP may lead to unpredictable long-term side effects.

In the spinal cord, the barrier between blood and neuronal tissue is formed by the blood-spinal cord barrier, and in the peripheral nervous system, the endothelial blood vessels and perineurium form the blood-nerve barrier. Drugs must pass through these barriers to the peripheral or central nervous system to modulate pain. In animals, altering the route of administration, such as direct delivery to the spinal cord or brain or, more subtly, by microinjecting drugs into the DRG, could help overcome these limitations; however, this is challenging in humans. Therefore, future studies on NP should focus on the underlying epigenetic mechanisms and tissue-selective drug delivery. In addition, whole-genome methylation assays are warranted. Epigenomic editing could enable the targeting of selected modifications for the design of individualized treatments for NP patients, ideally specifically targeting the affected cells (89, 90).

The role of gender is another important consideration. Indeed, clinical studies have shown that women are at higher risk of chronic pain and exhibit greater pain sensitivity. Sex hormones, endogenous opiate functions, and genetic factors are the main reasons for these differences (91–93). For example, in preclinical trials, male and female animals respond differently to pain under the same conditions (94); female CD-1 mice required two to three times more morphine than male mice to produce the same analgesic effect (95). Furthermore, the Delta opiate receptor (DOR) helps control pain, and pain tolerance was abolished in females with DOR knockout but not in male mice (96). In the context of epigenetics and gender, gender bias in DNA methylation levels has been found in many animal and human studies (97–99). Thus, as epigenetics can regulate opiate receptors, receptors display sex differences. Currently, the available evidence does not support gender-specific DNA methylation-based treatment of NP, but this is a conceivable future outcome. Further research is now needed to elucidate the underlying epigenetic and gender-based differences in pain response.

## Conclusions

Although an increasing number of studies have been conducted on NP animal models and DNA methylation, numerous promising therapeutics have failed in clinical trials (100, 101). The pain-causing gene or protein targets identified in animals may not be major contributors to pain in humans due to species differences in pain-modulation pathways. Basic cellular and molecular differences between animals and humans usually underlie these failures. Furthermore, there are also some uncertainties, such as the pathophysiological mechanism of pain in specific patients, dose selection in clinical trials, and the inability of animal models to accurately reflect the complex emotional responses humans have to pain (102, 103). In the context of difficulties in preclinical to clinical translation, improved animal models and a focus on pain circuitry are also needed to address epigenetic therapy. Given that NP remains a challenging condition to manage and the contribution of DNA methylation to this disorder is becoming increasingly recognized, the significance of DNA methylation in NP could become more apparent in coming years, and novel ways to study DNA methylation will likely be the primary focus of further studies.

## Author Contributions

WJ and X-YT designed the study, reviewed the literature, and drafted the manuscript. J-ML created the summary table and helped with the writing and revision of the manuscript. PY and MD contributed significantly to the revision of the final manuscript. All authors contributed to the article and approved the submitted version.

## Funding

This work was supported by grants from the National Natural Science Foundation of China (Grant No. 31872772) and the Natural Science Foundation of Jilin Province of China (Grant no. 20200201606JC) to MD.

## Conflict of Interest

The authors declare that the research was conducted in the absence of any commercial or financial relationships that could be construed as a potential conflict of interest.

## Publisher's Note

All claims expressed in this article are solely those of the authors and do not necessarily represent those of their affiliated organizations, or those of the publisher, the editors and the reviewers. Any product that may be evaluated in this article, or claim that may be made by its manufacturer, is not guaranteed or endorsed by the publisher.

## Abbreviations

BDNF: brain-derived neurotrophic factor
CCI: chronic constriction injury
CFA: Complete Freund's Adjuvant
CpG: cytosine-phosphate-guanosine
CREB: cAMP response element-binding protein
CSF1: colony-stimulating factor-1
DNMTs: DNA methyltransferases
DOR: Delta opioid receptor
DRG: dorsal root ganglion
GABA: γ-aminobutyric acid
HAT: histone acetyl transferase
HDACs: histone deacetylase family of enzymes
KOR: kappa-opioid receptor
MBD: methyl-CpG-binding domain
MDS: myelodysplastic syndromes
MeCP2: methyl-CpG-binding protein 2
MOR: Mu opioid receptor
miRNAs: MicroRNAs
NP: neuropathic pain
PFC: prefrontal cortex
TET: ten-eleven translocation
TFs: transcription factors
shRNAs: short hairpin RNAs
SNL: spinal nerve ligation
SNI: spared nerve injury
siRNAs: small interfering RNAs
5hmC: 5-hydroxymethyl cytosine
5mC: 5-methylcytosine.
